# Hypnotic relaxation results in elevated thresholds of sensory detection but not of pain detection

**DOI:** 10.1186/1472-6882-14-496

**Published:** 2014-12-15

**Authors:** Sybille Kramer, Rolf Zims, Michael Simang, Linda Rüger, Dominik Irnich

**Affiliations:** Multidisciplinary Pain Centre, Department of Anesthesiology, University of Munich, Munich, Germany

## Abstract

**Background:**

Many studies show an effectiveness of hypnotic analgesia. It has been discussed whether the analgesic effect is mainly caused by the relaxation that is concomitant to hypnosis. This study was designed to evaluate the effects of hypnotic relaxation suggestion on different somatosensory detection and pain thresholds.

**Methods:**

Quantitative sensory testing (QST) measurements were performed before and during hypnosis in twenty-three healthy subjects on the dorsum of the right hand. Paired t-test was used to compare threshold changes. The influence of hypnotic susceptibility was evaluated by calculating correlation coefficients for threshold changes and hypnotic susceptibility (Harvard group scale).

**Results:**

During hypnosis significantly changed somatosensory thresholds (reduced function) were observed for the following sensory detection thresholds: Cold Detection Threshold (CDT), Warm Detection Threshold (WDT), Thermal Sensory Limen (TSL) and Mechanical Detection Threshold (MDT). The only unchanged sensory detection threshold was Vibration Detection Threshold (VDT). No significant changes were observed for the determined pain detection thresholds (Cold Pain Thresholds, Heat Pain Thresholds, Mechanical Pain Sensitivity, Dynamic Mechanical Allodynia, Wind-up Ratio and Pressure Pain Threshold). No correlation of hypnotic susceptibility and threshold changes were detected.

**Conclusion:**

Hypnotic relaxation without a specific analgesic suggestion results in thermal and mechanical detection, but not pain threshold changes. We thus conclude that a relaxation suggestion has no genuine effect on sensory pain thresholds.

**Trial Registration:**

ClinicalTrials.gov, Identifier: NCT02261155 (9^th^ October 2014).

## Background

Hypnosis is one of the oldest treatment forms of pain. There is an increasing evidence of its effectiveness in the therapy of acute and chronic pain [1–6]. The question has been raised over whether (a) hypnotic analgesia is a unique pain reduction strategy, (b) a combination of different behavioral and cognitive elements, or (c) if the element of relaxation is responsible for an important part of the analgesic effect [7, 8]. This question cannot be answered easily since the relation between hypnotic analgesia and the different hypnotic techniques/suggestions seems complex [9]. The most common procedure in hypnotic analgesia is a hypnotic introduction which is followed by a relaxation suggestion. This forms the basis for further suggestions and is thus often referred to as "neutral hypnosis". Afterwards a specific focused analgesic suggestion is employed, (e.g. a glove of numbness being pulled over the painful extremity). The analgesic effectiveness of these different suggestions is not yet sufficiently clarified: Some studies found hypnotic relaxation to be equally effective as analgesic suggestions [10], whereas others showed that analgesic suggestions are more effective [8, 11, 12]. The consequence of this discussion might appear to only be existent in theory, as the combination of varying hypnotic techniques often results in the most effective form of pain relief. The size of this analgesic effect can be so powerful that even surgery with hypnosis as sole anesthesia has been described [13]. However there are studies indicating that not every individual might profit from every suggestion in a similar way: It seems that the individual characteristic of hypnotic susceptibility is essential regarding the ability to follow along more complex hypnotic suggestions such as focused analgesia. Hypnotic susceptibility or hypnotizability describes not only the ability to enter a hypnotic state but has also been shown to affect totally different functions as for example postural control [14]. A number of investigations could show that pain reduction was more effective in high than in low hypnotizable subjects [8, 15, 16] and it seems that at least in a non-hypnotic state only highly hypnotizable subjects can profit from specific suggestions [17].

Furthermore the extent of hypnotic analgesia can not only be influenced by the selected suggestion and the individual hypnotizability, but in an experimental setting it seems that not every type of stimulus can be modulated by hypnotic suggestions to the same degree as others [18]. One shortcoming of most existing studies is the lack of a comprehensive measurement of sensory modalities.

For a better understanding this study was planned to address the following considerations:

1. There are a lot of different hypnotic suggestions and techniques, but it is unclear if neutral hypnosis as described above has analgesic effects itself. Thus it was the main objective of this study to evaluate the modulating effect of neutral hypnosis on a certain stimulus. For a comprehensive evaluation of this effect quantitative sensory testing (QST) was chosen as a method. It is a well-established, standardized protocol evaluating different thermal and mechanical detection and pain thresholds [19, 20].
2. The individual hypnotic susceptibility seems to have an impact on effects achieved by hypnosis. It was further objective of this study to evaluate its influence on the observed results.

## Methods

### Subjects

Twenty-three healthy subjects aged 31.7 ± 2.8 (mean ± sem; 10 male, 13 female) participated in this study. All subjects participated voluntarily and gave written informed consent. The study was carried out according to the Helsinki Declaration, and was approved by the ethics committee of the Ludwig-Maximilian-University of Munich, Germany.

Excluded from the study were subjects with a history of major psychiatric disease, substance abuse, severe systemic, metabolic or neurological disease capable of influencing quantitative sensory testing.

### Design

In a single group pretest posttest design the effects of the hypnotic state on sensory parameters (quantitative sensory testing (QST)) were assessed. Hypnotic susceptibility was tested in all subjects before participation in the QST measurements. QST measurements were performed on the back of the right hand proximal to DII and DIII before and during hypnosis.

### Hypnosis and hypnotic susceptibility

Hypnosis and testing for hypnotic susceptibility were performed by a trained hypnotherapist.

Forty-nine Participants were tested for hypnotic susceptibility following the German norms of the Harvard Group Scale of Hypnotic Susceptibility, Form A [21]. Twenty-three subjects were selected for QST measurements depending on their level of hypnotic susceptibility. Four subgroups were formed depending on the achieved points in the Harvard Group Scale of Hypnotic Susceptibility: 0–3 points: low hypnotizability (LH), 4–6 low-medium hypnotizability (LMH), 7–9 high-medium hypnotizability (HMH), 10–12 high hypnotizability (HH). Descriptive data of the four subgroups is displayed in Table 1. Neither the participants nor the QST examiner, nor the hypnotherapist were informed about the results of the susceptibility testing.

**Table 1:** **Results for the different thresholds of quantitative sensory testing (QST) before (baseline) and during hypnosis (mean ± SEM)**

	Baseline	Hypnosis	***sign. (p-value)***
Thermal thresholds			
Cold detection threshold CDT (°C from baseline 32°C)	-1.57 ± 0.35	-5.09 ± 0.98	0.001*
Warm detection threshold WDT (°C from baseline 32°C)	2.81 ± 0.65	4.15 ± 0.69	0.002*
Thermal sensory limen TSL (°C)	3.63 ± 0.75	7.47 ± 1.01	0.000*
Paradox heat sensation PHS (x/3)	0.0 ± 0.0	0.0 ± 0.0	*--*
Cold pain threshold CPT (°C)	17.81 ± 1.85	17.98 ± 1.99	*0.90*
Heat pain threshold HPT (°C)	41.94 ± 0.80	41.30 ± 1.14	*0.53*
Mechanical thresholds			
Mechanical detection threshold MDT (mN)	2.01 ± 0.38	4.24 ± 0.87	0.000*
Mechanical pain threshold MPT (mN)	34.13 ± 6.99	44.37 ± 7.09	*0.04*
Mechanical pain sensitivity MPS	3.11 ± 0.82	2.15 ± 0.50	*0.01*
Dynamic mechanical allodynia DMA	0.05 ± 0.04	0.005 ± 0.00	*0.30*
Wind-up RatioWUR	2.68 ± 0.47	2.88 ± 0.41	*0.27*
Vibration detection threshold VDT (x/8)	7.61 ± 0.09	7.63 ± 0.12	*0.63*
Pressure pain threshold PPT (Pa)	246.27 ± 19.14	245.73 ± 19.91	*0.74*

*indicates statistical significance. The level of statistical significance (p-values) had to be adjusted for multiple testing because of the number of parameters that were evaluated (Bonferroni-Adjustment: significance level: p-values < 0.0041).

Hypnosis was verbally induced using the fixation method. The induction phase of the hypnotic state was standardized in its wording for all individuals. After a suggestion of palpebral catalepsy, the participants were asked to try to open their eyes as a manipulation check. If they stated that this was not possible, the hypnotherapist asked them to focus their attention on imagining an individual situation of well-being and calmness they had described before hypnosis. They were asked to imagine visual, auditory and tactile stimuli associated with the image in detail. The hypnotherapist then inquired about the situation and place the participants had reached in order to once more verify the hypnotic state. This individual part of hypnotic suggestion was repeated after each section of QST.

### Quantitative sensory testing (QST)

QST was performed following the protocol developed by the German Research Network on Neuropathic Pain (DFNS) to improve the diagnostic value of QST and provide a broad basis of reproducible results [19, 20].

### Thermal thresholds

Thermal testing was performed using a Peltier-based computerized thermal stimulator (TSA II; Medoc Inc., Ramat Ishai, Israel), with a 3 × 3 cm contact probe. All thresholds were measured using ramped stimuli (1°C/s) with a baseline temperature of 32°C. Cut-off temperatures were 0°C and 50°C. Cold and warm detection thresholds (CDT, WDT) were assessed, as well as paradoxical heat sensations (PHS) during thermal sensory limen procedure (TSL) of alternating warm and cold stimuli. Afterwards, cold and heat pain thresholds (CPT, HPT) were obtained.

### Mechanical detection thresholds

Mechanical detection thresholds (MDT) were assessed with a set of standardized von Frey filaments with forces two from 0.25 mN to 512 mN (Marstock-nervtest Ltd., Marburg, Germany). Using the "method of limits", five ascending and five descending series of stimuli were applied (1 s duration per stimulus).

### Mechanical pain thresholds

Mechanical pain thresholds (MPT) were measured with pinprick stimulators (non-injuring tip with a diameter of 0.2 mm) with fixed stimulus intensities from 8 mN to 512 mN (Department of Physiology and Pathophysiology, Mainz, Germany) [22]. Thresholds were calculated as the geometric mean of ascending/descending stimulus forces until the first perception/loss of sharpness.

### Stimulus-/response-function (SRF): mechanical pain sensitivity and dynamic mechanical allodynia

In a separate test, a stimulus–response function for the mechanical pain sensitivity (MPS) was determined using the same pinpricks already described to activate Aδ-nociceptors [22–24]. Additionally pain in response to light touch (dynamic mechanical allodynia; ALL) was tested by light stroking with a cotton wisp (3 mN), a cotton wool tip fixed to an elastic strip (100 mN) and a brush (200–400 mN). Each of the seven intensities of pinpricks and the three intensities of light stroking were applied five times in a randomized sequence. The subjects were asked to rate pain intensity of each stimulus on a numerical rating scale (NRS; 0 = no pain, 100 = maximal imaginable pain). The mechanical pain sensitivity was calculated as the geometric mean of all pain ratings for pinprick stimuli dependent on the applied intensity. Dynamic mechanical allodynia was quantified as the geometric mean of all numerical pain ratings after light touch stimuli.

### Wind-up Ratio (WUR)

The wind-up ratio (WUR) was examined using ten repetitive pinprick stimuli (1 Hz) compared to a single pinprick stimulus with a force of 256 mN. Wind-up ratio was calculated as the mean pain rating of five series of repetitive pinprick stimuli divided by the mean pain rating of five single stimuli.

### Vibration detection thresholds

Vibration detection thresholds (VDT) were examined with a Rydel-Seiffer tuning fork (64 Hz) that has a graded readout of vibration amplitude (from 0 to 8). Vibration detection thresholds were assessed by three series of descending stimulus intensities.

### Pressure pain thresholds

Pressure pain thresholds (PPT) were measured using a pressure algometer (FDK20, Wagner Instruments, Greenwich, CT, USA) with a range between 2 and 20 kg. The algometer had a rubber tip with a contact area of 1 cm^2^. The algometer was pressed to the skin with an increasing ramp of 0.5 kg/s, and the patient was asked to respond verbally as soon as the pressure became painful. This procedure was performed three times.

### Data analysis

All data are presented as raw data (mean ± SEM). For statistical analysis several QST variables (CDT, WDT, TSL, MDT, MPS, ALL, WUR and PPT) were transformed logarithmically as recommended by Rolke et al., [20] resulting in normally distributed variables. To prevent the loss of zero values, 0.001 was added to zero before the data transformation. Hence, the pre-post comparison was performed by a paired sample t-test. Because of the varity of QST measures an α-adjustment for mulitple testing according to Bonferroni was carried out with a p-value < 0.0041 regarded as statistically significant.

The identification of possible outcome differences depending on the hypnotic susceptibility was calculated by an ANCOVA as suggested by Vickers [25]. Data preparation and all calculations were performed by using the statistical package for social sciences (IBM SPSS 19 for Windows).

## Results

Quantitative sensory testing results from the dorsum of the right hand from twenty-three healthy subjects before and during hypnosis were compared. One subject had to be excluded from analysis because of falling asleep during the second QST procedure.

### Somatosensory profile

**Figure 1:**
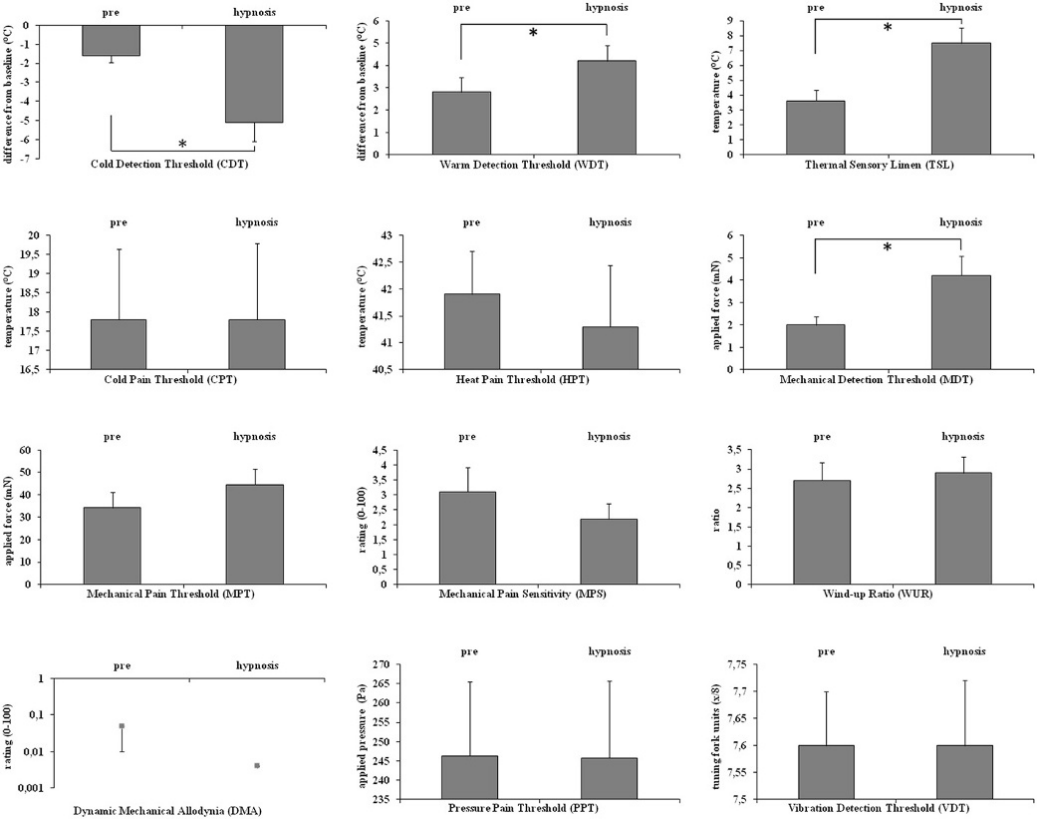
**Quantitative sensory testing (QST) thresholds before and during hypnosis.** *indicates statistical significance.

Results of quantitative sensory testing are shown in Table 2 and Figure 1.

**Table 2:** **Descriptive data of quantitative sensory testing (QST) thresholds before (pre) and during hypnosis according to individual hypnotic susceptibility**

	LH pre hypnosis	LH during hypnosis	LMH pre hypnosis	LMH during hypnosis	HMH pre hypnosis	HMH during hypnosis	HH pre hypnosis	HH during hypnosis
Patients per group (n total = 22)	n = 3	n = 3	n = 8	n = 8	n = 4	n = 4	n = 7	n = 7
Thermal thresholds								
Cold detection threshold CDT (°C from baseline 32°C)	-1.3 ± 0.34	-3.0 ± 1.38	-0.98 ± 0.12	-2.7 ± 0.61	-2.3 ± 1.44	-9.3 ± 2.44	-1.95 ± 0.76	-6.3 ± 2.16
Warm detection threshold WDT (°C from baseline 32°C)	5.5 ± 3.65	3.6 ± 1.01	1.5 ± 0.37	2.5 ± 0.35	2.5 ± 1.47	4.3 ± 1.13	3.3 ± 0.98	6.3 ± 1.79
Thermal sensory limen TSL (°C)	7.5 ± 4.18	6.8 ± 1.40	2.2 ± 0.45	4.4 ± 1.20	2.9 ± 1.59	9.5 ± 2.15	4.1 ± 1.07	10.1 ± 2.06
Paradox heat sensation PHS (x/3)	0.0 ± 0.0	0.0 ± 0.0	0.0 ± 0.0	0.0 ± 0.0	0.0 ± 0.0	0.0 ± 0.0	0.0 ± 0.0	0.0 ± 0.0
Cold pain threshold CPT (°C)	12.7 ± 3.48	12.9 ± 3.41	18.3 ± 2.99	21.7 ± 2.58	18.2 ± 5.40	10.8 ± 5.02	19.2 ± 3.70	20.0 ± 3.95
Heat pain threshold HPT (°C)	46.3 ± 1.78	42.3 ± 1.58	40.3 ± 0.90	39.2 ± 2.09	43.8 ± 1.00	41.3 ± 3.71	40.9 ± 1.69	43.2 ± 1.65
Mechanical thresholds								
Mechanical detection threshold MDT (mN)	1.9 ± 0.82	5.2 ± 2.58	2.2 ± 0.66	3.1 ± 0.88	3.4 ± 1.28	5.0 ± 1.40	1.0 ± 0.24	4.7 ± 2.35
Mechanical pain threshold MPT (mN)	35.3 ± 10.08	73.6 ± 11.78	29.8 ± 14.29	26.6 ± 6.38	40.3 ± 15.06	48.3 ± 7.25	35.0 ± 13.39	49.9 ± 18.35
Mechanical pain sensitivity (MPS)	1.2 ± 0.43	0.8 ± 0.24	3.0 ± 1.54	1.8 ± 0.71	3.4 ± 1.46	2.1 ± 0.96	3.9 ± 1.82	3.2 ± 1.21
Dynamic mechanical allodynia (DMA)	0.0 ± 0.03	0.0 ± 0.01	0.0 ± 0.01	0.0 ± 0.00	0.0 ± 0.00	0.01 ± 0.01	0.1 ± 0.13	0.01 ± 0.01
Wind-up (WUR)	1.4 ± 0.23	1.8 ± 0.10	3.7 ± 1.07	4.2 ± 0.95	3.3 ± 0.84	2.59 ± 0.22	1.7 ± 0.45	2.0 ± 0.26
Vibration detection threshold (VDT) (x/8)	7.3 ± 0.38	7.8 ± 0.22	7.6 ± 0.16	7.7 ± 0.15	7.9 ± 0.08	8.0 ± 0.00	7.6 ± 0.16	7.3 ± 0.28
Pressure pain threshold (PPT; Pa)	245.0 ± 54.20	261.7 ± 67.43	267.0 ± 37.54	265.3 ± 33.52	318.0 ± 30.53	327.0 ± 29.00	182.1 ± 14.38	170.1 ± 17.28

Subjects were divided into the following subgroups: low hypnotizable subjects (LH), medium-low hypnotizable subjects (MLH), medium-high hypnotizable subjects (MHH) and high hypnotizable subjects (HH). Data is presented as mean ± SEM.

Cold detection thresholds (CDT) were significantly lowered, warm detection thresholds (WDT) were significantly elevated during hypnosis compared to the measurements before hypnosis. Accordingly, Thermal sensory limen (TSL), assessed by alternating CDTs and WDTs, were significantly elevated. No Paradoxical heat sensations (PHS) (assessed during TSL procedure) were observed before or during hypnosis. Cold pain thresholds (CPT) and Heat pain thresholds (HPT) did not show significant changes.

Mechanical detection thresholds (MDT) were significantly increased during hypnosis. Mechanical pain thresholds (MPT) and Mechanical pain sensitivity (MPS) showed a trend towards an increased threshold. It did not reach statistical significance due to Bonferroni-adjustment for multiple testing that resulted in a lowered significance level of p < 0.0041 instead of the usual 0.05. Dynamic mechanical allodynia (DMA), Wind-up ratio (WUR), Vibration detection thresholds (VDT) and Pressure pain thresholds (PPT) did not show significant changes.

**Figure 2:**
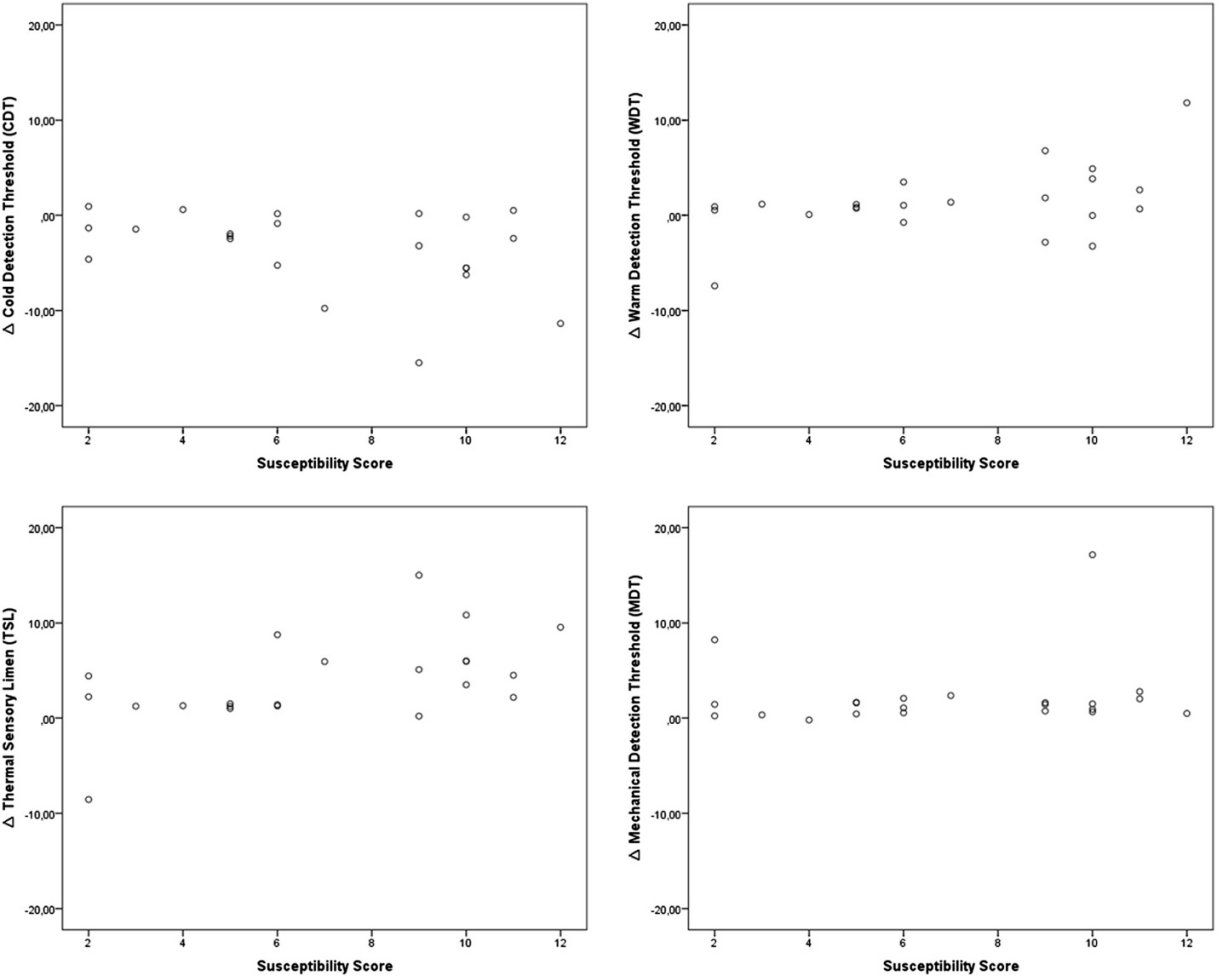
**Distribution of the significantly changed somatosensory thresholds during hypnosis dependent on the hypnotic susceptibility level (achieved points in the Harvard Group Scale Test).** No correlation of the hypnotizability to the threshold changes was observed.

No significant influence of hypnotic susceptibility on sensory thresholds changes was observed (Figure 2). Results of QST in the different hypnotizability groups are shown in Table 1.

## Discussion

In this study a significant change (reduced function) of different sensory detection thresholds (WDT, CDT, TSL, MDT) during hypnosis was observed. Vibration detection threshold (VDT) and all pain thresholds (CPT, WPT, MPT, MPS, DMA, WUR and PPT) did not show statistically significant changes. A correlation of sensory threshold changes with the determined hypnotic susceptibility score could not be demonstrated.

The observed results indicate that hypnosis without a specific analgesic suggestion has no influence on pain thresholds, independent of the modality that is the source of pain (thermal, mechanical, etc.). It is the strength of this investigation that it evaluates the effect of hypnosis on sensory perception in a battery of standardized sensory tests such as quantitative sensory testing (QST). Not only does QST offer the possibility to determine the amount of sensory loss and small fibre inhibition, but it can also provide information about pain perception and the cerebral processing of nociceptive data [26]. In respect of our results the latter is the more important function: The threshold changes induced by hypnosis in this study cannot be related to a certain type of fibres or spinal pathways, as they do not match a pattern of congruency for Aδ-, Aβ-and C-fibre-affection. This shows that hypnosis does not specifically affect one kind of peripheral afferent nerve fibre but has an impact on central processing of perception. From among the various kinds of kinds of central modulation of pain perception, one possibility is distraction. Our findings, however, do not allow a definitive conclusion. Nevertheless a further hint that the main reason for the observed reduced functions in detection is distraction is: Out of all somatosensory detection thresholds evaluated in our study, only vibration detection threshold (VDT) showed no change during hypnosis. VDT is the only detection threshold in QST which is determined by a stimulus starting with full intensity decreasing to zero instead of a stimulus starting at zero and increasing intensity. The proband is more likely to immediately focus on a full intensity stimulus than on a stimulus that slowly reaches the individual perception threshold.

In consideration of the above said, the order of stimuli (pain vs nonpain) might have had an impact on the observed results. Painful stimuli might have resulted in a higher awareness for the testing of the next non-painful stimuli. To avoid this the individual part of the suggestion was repeated after each section.

Our study was designed to improve basic physiological understanding of the influence of relaxation on sensory thresholds. Therefore the transfer of our findings into clinical practice is limited. For a definitive conclusion on the role of relaxation as a part of hypnotic analgesia, a comparison of procedures with and without the element of relaxation is needed.

Furthermore the assessment of the subjectively perceived relaxation or an objective measurement of physiological parameters should be included, as well as the assessment of individual centeracteristics that might have influenced pain thresholds. Another limitation is the low sample size because of which we might have missed minor effects. On the other hand we believe that the sample size was enough to detect clinically relevant effects.

### The role of the stimulus

Most previous investigations concentrated on evaluating the analgesic effect of a hypnotic suggestion on a certain type of pain stimulus. However, we found one investigation that compared the effects of three different suggestions and placebo on electric stimuli [27]. The detection and pain thresholds in their investigation could correspond to the mechanical detection threshold (MDT) and the mechanical pain threshold (MPT) in our study, only the source of the stimulus was different (electric pain stimulus). Our results confirm the observation that pain thresholds were not significantly altered by relaxation suggestions. But in contrast to our results they did not observe a significant effect of relaxation on detection thresholds. It has been hypothesized before [17] that the type of applied stimulus influences the result. This might be the reason for this discrepancy.

#### The role of the hypnotic suggestion

There are a number of investigations that found significant changes of pain thresholds in a clinical setting. These publications seem to be contradictory to our findings, but they all employed specific analgesic suggestions [15, 28, 29] in contrast to the relaxation suggestion that was used in our investigation. It has been shown previously that rather the type of hypnotic suggestions than hypnosis itself influences the perception and processing of sensory and pain stimuli [16] with an advantage of a specific analgesic suggestion regarding pain relief. More insight could be gained by comparing the effect of different suggestions.

### The role of the affective dimension of pain

It has been shown that hypnosis in general, but especially hypnotic relaxation influences the affective dimension of pain perception to a larger extent than the sensory dimension [29]. Furthermore emotion and attention can have a differential effect on pain [30]. In our study we used quantitative sensory testing to evaluate the different thresholds. QST aims at the sensory component of perception rather than on affective aspects. Therefore, even if our results are in contrast to the observation that hypnotic relaxation has an analgesic effect in a clinical setting [7, 31], in the above described context, our findings still indicate that a hypnotic relaxation suggestion does not unfold a specific effect on pain perception. Future studies might need to introduce a parameter to assess affective aspects in addition to evaluating sensory thresholds.

### The role of hypnotic susceptibility

A correlation of the individual hypnotic susceptibility with the analgesic effect has been described for a specific analgesic suggestion with a number of investigations showing that pain reduction was more effective in high than in low hypnotizable subjects [8, 15, 16, 32, 33], even without prior induction of a hypnotic state [17, 34]. For unspecific effects caused by general effects of hypnosis without specific suggestions the individual susceptibility does not seem to be of importance [15]. We believe that the type of suggestion is the reason that we did not find a correlation of the susceptibility score and sensory changes in our study. This observation in the context of the above mentioned result in literature has an important implication for the clinical practice: Even if a relaxation suggestion has no genuine analgesic effect it might still be effective in a clinical setting, due to distraction and a modulation of the affective component of pain. Furthermore, especially in low hypnotizable subjects who do not manage to follow along a specific analgesic suggestion it might even turn out to be equally effective. To proof this assumption future study should focus on the influence of different suggestions on somatosensory threshold changes as a function of hypnotic susceptibility and the proportion of sensory/affective components of pain.

## Conclusion

In summary our findings show that the induction of a hypnotic state without special analgesic suggestions has no effects on pain perception thresholds but solely leads to an increase in sensory detection thresholds. The analgesic effect of hypnosis without specific suggestions in clinical studies thus seems to be caused by distraction and a modulation of the affective component of pain. These effects do not depend on hypnotic susceptibility. However, it is known that the intensity of pain relief can depend on the individual susceptibility as can the analgesic effect of specific suggestions. Therefore further investigations should introduce a parameter to assess affective modulation of pain and compare the effect of different suggestions on somatosensory thresholds according to the hypnotic susceptibility.
